# Striving for Career Establishment: Young Adults’ Proactive Development Under Career Identity and Passion Dynamics

**DOI:** 10.3390/bs15101402

**Published:** 2025-10-15

**Authors:** Peter Yang

**Affiliations:** 1 Department of Counseling, National Chiayi University, Chiayi 621, Taiwan; p.yang71@yahoo.com; 2 Institute of Sociology, Academia Sinica, Taipei 11529, Taiwan

**Keywords:** adaptation, agency, career identity, career passion, career striving, proactive behavior

## Abstract

This study aimed to provide a comprehensive understanding of career striving by exploring the trajectory of career identity and passion, particularly focusing on the evolution of young people’s self-direction and energy while establishing their vocational careers. Utilizing the interpretative phenomenology paradigm, semi-structured in-depth interviews were conducted with 30 university graduates who had been employed for approximately three years since entering the workforce. Data were analyzed using a modified form of interpretative phenomenological analysis, combining idiographic depth with cross-case thematic synthesis. The analysis identified 20 experiential themes that captured critical aspects of career striving, including the career growth model, stress-coping model, and associated mechanisms. The conceptualization of career striving established in this study provides a theoretical framework for the development of career striving theory and implications for further research.

## 1. Introduction

In the context of a rapidly evolving labor market, young workers are increasingly confronted with multifaceted challenges, including occupational socialization, skill mismatch, identity confusion, and work adjustment difficulties (Sullivan & Al Ariss, 2021; Wong & Mohd Rasdi, 2019). These challenges are compounded by globalization, automation and digitalization, and shifting societal expectations, all of which contribute to heightened uncertainty and competitiveness in career development (Yeung & Yang, 2020). The emergence of unstable career trajectories, fragmented employment histories, and nonlinear transitions into the labor market demands a shift in focus from traditional career development models to more dynamic and proactive approaches (Klehe et al., 2021; Langerak et al., 2022). In light of this context, proactive career management has become essential for young workers transitioning from higher education to the workforce (Hirschi & Koen, 2021; Hirschi & Pang, 2025; Hirschi et al., 2022; Ivaldi et al., 2022; Medici et al., 2023). Furthermore, young workers’ efforts to establish vocational careers are not influenced merely by their preparedness to enter the workplace through higher education, but also by their adaptation to the work environment in the early years of their work life (Klehe et al., 2021; S. Wang et al., 2021). However, little is known about how young workers can behave proactively to establish their careers in a dynamic and ever-evolving contemporary workplace. Their career management and development throughout their higher-education-to-work transitions, as well as the underlying dynamics in their early work years, remain unclear.

This study introduces the concept of career striving as a critical phenomenon in understanding how young workers actively construct and advance their career establishment amid external and internal challenges. Career striving refers to the proactive and persistent pursuit of career-related goals, characterized by an individual’s efforts to overcome contextual and personal constraints (Creed et al., 2013; Dik et al., 2008). This construct is situated at the intersection of career motivation and proactive career behavior, highlighting the interplay between intrinsic motivation and agentic career action. Rather than adopting a reactive stance, individuals engaged in career striving exhibit forward-looking, self-initiated behaviors that align with long-term career objectives. These behaviors are energized and guided by two key motivational mechanisms: career identity, which provides direction, and career passion, which sustains engagement and resilience (Ali Abadi et al., 2023; Jiang et al., 2021; Meijers & Lengelle, 2012; O’Keefe et al., 2022). The two dimensions form the motivational engine that propels individuals to invest in and commit to their career goals, even in the face of setbacks and uncertainty.

The theoretical significance of career striving lies in its potential to enrich existing career construction theory (Fu et al., 2023; McIlveen & Luke, 2023; Savickas, 2020; D. Wang & Li, 2024) and proactive behavior theory (Akkermans & Hirschi, 2023; Chang et al., 2023; Jiang et al., 2023) by emphasizing motivation as a dynamic, multi-dimensional, and context-sensitive process. While career construction theory emphasizes adaptability, narrative identity, and the co-construction of career meaning, it pays limited attention to the energizing role of passion and its interaction with identity during key transitional phases. Similarly, proactive behavior theory has primarily focused on observable agentic behaviors such as career planning and networking, without clearly examining the motivational substrates that drive such behaviors. By positioning career identity and career passion as foundational yet distinct motivational forces, this study seeks to bridge the gap between these two theoretical paradigms and provide an integrative model of career self-management.

There is a critical need for an integrated framework that captures the temporal and motivational dynamics underpinning proactive career development. This framework must account for how young workers experience, reflect upon, and respond to career-related feedback, constraints, and opportunities over time. Understanding how career identity and passion interact to support persistence, adaptability, and agency can enhance theoretical explanations of proactive behavior and contribute to the design of more responsive Human Resource Development (HRD) interventions.

To address these gaps, this study addresses the following research questions:

1. How does career striving evolve between the pre-graduation career preparation and workforce entrance stages, and then the early years of working?
2. During career establishment, what mechanisms determine the trajectories of career striving?

A dual-dimensional framework centered on career identity and passion is established in this study to analyze the proactive efforts of young workers during career establishment. Drawing from Meijers’ (1998) “person in context” perspective and contemporary conceptualizations of career passion (O’Keefe et al., 2022), this framework elucidates how identity provides strategic direction and passion fuels sustained effort. Career identity provides a sense of coherence and direction by aligning individuals’ self-concept with occupational roles and future aspirations (Ali Abadi et al., 2023; Meijers & Lengelle, 2012; Wendling & Sagas, 2024). Career passion, on the other hand, provides the emotional and motivational intensity that drives continued engagement, persistence, and resilience (Jiang et al., 2021; Lotz et al., 2025; O’Keefe et al., 2022). By integrating these constructs within the broader context of proactive behavior, the study provides an in-depth understanding of the motivational mechanisms underlying career striving.

This integrative approach allows for a more dynamic understanding of how young workers construct their careers in response to evolving circumstances. For example, a well-developed career identity may help individuals interpret workplace challenges as opportunities for growth, while sustained career passion may buffer the negative effects of early-career setbacks. Together, these dimensions provide a robust explanatory model for how young adults mobilize internal resources (Hirschi & Pang, 2025; Retkowsky et al., 2023) to manage the complexities and pressures inherent in contemporary work life.

The present study contributes to HRD theory by advancing a holistic model of career striving that captures both the behavioral and motivational dimensions of early career development. Specifically, it extends career construction theory by embedding passion as a central motivational resource that complements and reinforces identity development. It also expands proactive behavior theory by unpacking the internal drivers of agency, thus moving beyond behavioral outcomes to include underlying psychological processes.

Moreover, this study responds to the need for empirical evidence on how young workers actively manage career establishment in a highly dynamic and unpredictable labor market. Through qualitative inquiry, it illuminates how career striving is experienced and sustained in real-life contexts. The findings provide practical implications for career counseling, organizational management, and educational interventions, particularly in designing programs that support identity formation, cultivate passion, and strengthen proactive behaviors. By clarifying the mechanisms that determine career striving, practitioners and policymakers can design effective interventions to support young workers, including training programs that foster career identity and passion, as well as resources that promote proactive career behaviors (Hirschi & Pang, 2025). Educators may also benefit by aligning career counseling interventions with the dual components of striving: purpose-driven direction and passion-fueled persistence.

This study aims to strengthen theoretical and applied knowledge in the fields of career development and HRD by illuminating the mechanisms that enable young workers to thrive in contemporary career landscapes. In doing so, it not only fills important theoretical gaps but also provides insights for stakeholders invested in supporting early career success.

## 2. Method

### 2.1. Research Paradigm

This study adopted a qualitative research design grounded in interpretative phenomenology (IP), aiming to explore the lived experiences of young workers as they established their careers from higher education to the workplace. Semi-structured, in-depth interviews for data collection were employed to capture the depth and complexity of individual experiences, aligning with the recommendations from previous research on studying complex phenomena (Huff et al., 2019; Seidman, 2013). The primary focus of the interviews was obtaining rich and comprehensive information by establishing trust relationships, which was the basis for exploring the trajectories of career striving that unfold throughout the higher-education-to-work transition. Furthermore, this study examined the psychological dynamics involved in the initiation and manifestation of career identity and passion. It aimed to understand the evolution of proactive career striving, which emerges from the supportive interplay of career identity (providing direction) and career passion (providing energy) as young workers encounter various work- and career-related challenges.

To gain deep insights into individuals’ experiences and career striving, this study employed interpretative phenomenological analysis (IPA), emphasizing an idiographic approach (Eatough & Smith, 2017). This approach allowed for a thorough exploration of how young workers, in their unique contexts, interpreted and made sense of their situations, aligning with recommendations from previous IP research (e.g., Huff et al., 2019) on studying complex phenomena during the early phases of an investigation. Grounded in IP, the research process was reflexive and recognized the co-construction of meaning between researcher and participants through the double hermeneutic process (Giddens, 1986). The researcher’s professional background in counseling and career development informed this interpretative stance, allowing sensitivity to participants’ lived experiences of forming and sustaining career identity and career passion. By integrating IP and IPA, the study provided a coherent methodological framework for uncovering how identity provided direction and passion supplied energy in shaping proactive career striving.

### 2.2. Participants

Participants were selected based on specific inclusion criteria: being university graduates who had worked full-time for approximately three years post-graduation. To ensure diversity and comprehensiveness, participants were recruited from various sectors, including technology, finance, healthcare, education, and manufacturing. A total of 30 participants were included, ensuring a balanced mix of gender, regional background, university major, and occupational field. This diverse participant pool provided a robust basis for exploring career expectations and experiences.

The sample size of 30 participants was guided by the principle of interpretative saturation, ensuring sufficient depth and variation to capture experiential richness rather than numerical representation. This number is consistent with qualitative research standards in both IPA and thematic analysis. While recommended sample sizes for phenomenological studies typically range from 5 to 25 participants, and thematic analysis studies often range between 12 and 20 participants (Ahmed, 2025; Hennink & Kaiser, 2022), this study extended data collection to 30 interviews to ensure depth and saturation across a range of career domains. The final five interviews yielded no new themes, indicating that saturation had been reached (Hennink & Kaiser, 2022; Tight, 2024). This result further supports the adequacy and trustworthiness of the sample. Although participants represented diverse occupational backgrounds, the analytical focus remained on shared developmental processes of career striving rather than on domain-specific variations.

### 2.3. Procedure and In-Depth Interviews

Participants were recruited through department website announcements and voluntarily agreed to participate in in-depth interviews. The interview process aimed to establish a trusting rapport and gather comprehensive information on their career experiences. Interviews were semi-structured, allowing participants to freely share their narratives while ensuring key topics were covered, such as career decisions and preparations before graduation, transition experiences from education to work, current career developments and challenges, self-perception and identity at work, and career aspirations and future goals. Interviews typically lasted between 1.5 and 2 h, with participants encouraged to reflect on their career trajectories and how their experiences shaped their career striving.

The interview guide was developed based on existing literature (e.g., Creed et al., 2013; Dik et al., 2008) and refined through a trial interview. Expert consultation played a crucial role in shaping the guide. Experts included university professors with extensive research experience in HRD and career counseling practitioners. Their feedback ensured the relevance and comprehensiveness of the questions. The interview guide can be found in Appendix A. Sample questions included “Can you describe your career goals during your final year of university?”, “What were your experiences transitioning from university to your first full-time job?”, “Can you provide an example of a proactive step you took to advance your career?”, and “How do you perceive your career identity now compared to when you first graduated?”

At the end of each interview, the participants were given the opportunity to reflect on the interview session. They were encouraged to provide feedback on the proactive nature of their own career management, summarizing the main features of their career striving, discussing how it evolved between the higher education and work periods, and explaining why the direction and energy of their career striving persisted throughout their transition into the workplace.

### 2.4. Data Analysis

Data were analyzed using interpretative thematic analysis guided by IPA principles (Eatough & Smith, 2017; Guest et al., 2012; Huff et al., 2019). This approach provided a systematic means of identifying and interpreting recurring patterns within qualitative data, allowing for the emergence of descriptive yet meaningful themes grounded in participants’ narratives. Guided by the IP principles, the analysis extended beyond surface-level description to explore the subjective meanings participants attached to their experiences. This interpretative emphasis aligns with the phenomenological orientation of the study, which aims to understand how individuals make sense of their lived experiences and how these meanings shape their career development trajectories.

To accommodate the relatively large and diverse participant group, this study adopted a modified form of IPA that combined idiographic depth with cross-case thematic synthesis. While remaining grounded in the IP principles and emphasizing lived experience, reflexivity, and the double hermeneutic, the analysis moved iteratively between within-case interpretation and the identification of shared experiential patterns across cases. This approach preserved the richness of individual meaning-making while allowing broader interpretative insights into the common psychological and motivational dynamics underlying proactive career development.

To enhance methodological rigor, a three-stage thematic coding structure was adopted, consistent with IP principles. The IPA began with initial coding, where data were segmented into experiential meaning units that captured participants’ lived experiences and reflections. This stage was followed by focused coding, which involved identifying and interpreting recurring experiential patterns within and across cases. In the final integrative stage, broader experiential themes were developed to represent shared meanings while remaining closely aligned with participants’ language and narratives. This iterative and interpretative process maintained analytic depth and coherence, ensuring that the emerging themes reflected both the individuality of participants’ sense-making and the shared experiential structures of proactive career striving.

The overall analytic process included familiarization with the data, systematic interpretative coding, and the development and refinement of experiential themes. Each theme was illustrated with representative participant quotes to preserve individual voices and contextual depth. This iterative and interpretative process provided an in-depth understanding of participants’ experiences of career striving and illuminated how they made sense of their vocational development trajectories.

In addition, open conversations with each participant allowed for an in-depth exploration of their experiences, as well as for clarifying the themes. These qualitative data, rich in personal meanings and significance attributed to their career journeys, were analyzed using exploratory thematic analysis. Consequently, the research team gained insights into the features of the participants’ career striving experiences and how these experiences shaped their understanding of their own career trajectories.

Although the participants came from diverse occupational sectors, including education, healthcare, finance, and technology, the analysis concentrated on the shared developmental processes observed across cases. Rather than focusing on sector-specific themes, the study emphasized generalizable psychological mechanisms and motivational patterns involved in proactive career development. This inclusive, cross-context focus enhanced the relevance and applicability of the findings.

### 2.5. Multiple Strategies to Enhance Trustworthiness and Interpretative Rigor

To strengthen the credibility and interpretative rigor of the findings, multiple strategies were employed. These strategies included data source variation, methodological complementarity, analyst collaboration, and peer debriefing. Data source variation captured diverse perspectives on career striving across occupational contexts. Methodological complementarity involved follow-up interviews and participant feedback to refine and enrich emerging interpretations. Analyst collaboration engaged multiple researchers in reviewing data and developing themes to enhance interpretative coherence. Peer debriefing provided feedback from experts in career development and HRD to improve transparency and reflexivity. Together, these strategies supported a rigorous and reflexive analytic process, strengthening the trustworthiness and interpretative integrity of the interpretations.

## 3. Results

Twenty themes were developed from the data, representing the critical elements of career striving for young workers. In this paper, the results are first presented in terms of career identity and passion development (three themes for each), using them to explain how career striving evolves throughout the higher-education-to-work transition as well as during early work life (see Table 1). They are followed by a presentation of the psychological mechanisms underlying the trajectories of career striving and its proactive nature (seven themes for the Career Growth Model and seven themes for the Stress-Coping Model). Each theme is described and illustrated using quotes from the interview narratives.

### 3.1. Providing Direction for Career Striving: Career Identity Evolving from Career Decisions Made During Higher Education

The themes related to career identity highlight how decisions made during higher education shape career trajectories as individuals enter the workforce. Many participants found that their university career exploration did not lead to a focused path, resulting in job mismatches and necessary adjustments. As they gained work experience, they developed a clearer career identity, reconciling ideal aspirations with workplace realities, which led to effective career choices and growth. These narratives highlight the dynamic nature of career identity as young workers navigate early career challenges and seek to align their professional roles with personal goals.

#### 3.1.1. Theme #1: Career Direction Is Still Being Explored During University and Continues into Early Work Life

Many of the 30 participants’ attempts and experiences during university were reflected in the diverse career exploration activities they undertook. This multi-year career exploration process provided numerous opportunities for them to understand themselves and the external job environment, including through courses, club activities, or industrial-academic collaboration opportunities [C2, D5, E1]. However, these higher education experiences did not seem to constitute a “focused” career exploration [e.g., A3]. This lack of focus was reflected in an interview with one of the participants, as shown below.

Interviewer: So, were you thinking about your future career during your early university days?

Participant: Yeah, from the start I didn’t wanna waste those years of studying, but now that I’m out in the real world, things aren’t exactly going as planned. Ended up in a job with no skills, so now I’m hustling to switch careers [F2-9-2].

The long career exploration process seemed to continue from university to the early phase of some participants’ vocational career establishment, as they searched for jobs that fit their interests and abilities (Curşeu et al., 2021; Stremersch et al., 2021). In contrast to what they had previously experienced in higher education, direct and hands-on work experience helped them quickly develop a clear sense of their career identities.

#### 3.1.2. Theme #2: Pre-Professional Identity Evolves as University-Acquired Knowledge and Skills Are Applied in the Workplace

Many participants mentioned a strong connection between what they studied at university and their current jobs. Using early career identity (i.e., pre-professional identity, Jackson, 2016), they were able to secure job opportunities that matched their expectations. Although the job content was not always satisfying [F1, E3], they were able to apply their professional knowledge and pre-professional identities gained during university and strengthen their career identities at work. This occupational socialization process, which involves pre-professional socialization and identity transformation, also allowed them to gain more knowledge regarding their work and better understand and define themselves in their professional roles [D4, F3].

Apart from recognizing their strengths and limitations [C1, E5], the young workers also became clearer about what they hoped to achieve in their jobs. It was not just about making a career decision, like in university, such as whether to take an opportunity to obtain a specific job in the future. [A5].

*You know when I really figured out my teaching philosophy and what kind of teacher I wanted to be?…… It was when I started working! During internships, I was just listening to what teachers said, not really doing much. But once I started working and dealing with different children, it pushed me to develop my own unique abilities. Then, my teaching philosophy was crafting [D2-12-4].*

#### 3.1.3. Theme 3: Career Identity Crystallizes Through Compromise Between Ideals and Workplace Reality

In contrast to those who had already decided on a career direction in higher education, most participants experienced their career identity slowly after entering the workplace and facing a variety of work and career choices in a real work environment [B2, E4]. They began to understand and confirm what they truly want in their careers and aspire to achieve [B4, F5]. An example is as follows:

Interviewer: [Did] you figured out how to express yourself at work during these months?

Participant: Yeah, kinda. I show different sides of myself when dealing with different people. It’s like acting, you know? Similar to my major and what I’ve learned in drama, I feel like I’m putting on a show every day [B4-6-1].

Before entering the workplace, career self-concept and choices may not always align with the practical realities of starting a career, leading to feelings of disconnection after graduation and the need to start a new career [C3]. There is a clear gap between higher education and the work environment for young workers. They should strive to not only survive but also thrive in the workplace and establish a career identity that can guide their ongoing development.

### 3.2. Fueling Career Striving: Passion and Energy Strengthened for Actualizing the Ideal Career During Career Establishment

The themes reveal that young workers’ career passion originates from idealized visions formed during higher education, serving as motivational forces in the workplace. This passion, shaped by educational experiences and personal aspirations, is clear in the majority of participants’ enthusiastic career descriptions. As they begin their careers, practical experiences and early successes at work further fuel this enthusiasm, while a desire to overcome past constraints emphasizes the importance of autonomy. This evolution of career passion highlights the interplay between idealization and practical realization in shaping career striving.

#### 3.2.1. Theme #4: Career Passion Originates from an Idealized Career Vision During Higher Education

Regarding adapting to the work environment, there was significant consistency before and after entering the workplace, whether it involved adopting passive or active responses [C4, B5]. How young workers perceived the relationship between themselves and their previous studies and living environment in higher education determined their career passion. This process, known as career crafting (Akkermans & Tims, 2017; Ge et al., 2023), contributes to differentiating their development amidst the uncertainties of contemporary career and work-related challenges. The evolution of this motivational mechanism can be explained by understanding how an ideal career, previously established in higher education, transforms into career passion in the workplace.

When asked about how they envisioned entering the workplace in the past, many participants recalled that they were highly enthusiastic before graduation, describing their ideal career concretely and with much imagination about their imminent entry into the workplace [e.g., C5].

*Throughout the journey from university to the present, my goal has always been to get into the tech industry and have the opportunity to work abroad. I wanted to explore the world outside. I was passionately driven to conquer new horizons and blaze a trail beyond the boundaries of my current career! [E5-4-7].*

#### 3.2.2. Theme #5: Practical Success Reinforces the Pursuit of Ideal Career Goals

During the initial phase of career establishment, fulfilling personal goals aligned with expectations fostered a sense of achievement, thereby instilling new enthusiasm for work. Such experiences often stemmed from the pre-graduation interview process, wherein submitting resumes, attending interviews, and subsequently being selected provided young workers with opportunities to realize their envisioned career paths and accrue successful experiences. At this time, their fervor to pursue aspirational goals and ideal careers was truly ignited.

*But you know, during the interview process, I didn’t feel like I presented myself in the best way, and that really got me feeling down for a while… However, things took a turn for the better later on. I was fortunate enough to get in touch with the product engineer I admired the most back in high school, and it felt like a stroke of luck! [E5-3-4].*

Apart from adapting to work, participants believed in investing time to accumulate workplace experience. Facing career challenges head-on and seeking feedback were crucial in driving their inner motivation to pursue their career goals continuously.

#### 3.2.3. Theme #6: Striving Is Fueled by a Desire to Break Free from Past Limitations

Many other participants found strength in rebelling. For some, this rebellion was driven by a desire to effect personal change from their past selves or break free from stagnant life situations [B5, D2]. Others were motivated to escape family expectations and the confines of their upbringing, refusing to settle for a life that did not align with their true aspirations [D4]. They often recalled their youthful determination to do something they would not regret in the future, pushing themselves to work hard to achieve their goals [A1, B5].

*Yeah, back before I graduated, I had this big urge to spice up my life, you know? I didn’t want it to be all too ordinary. Coming from the city and being sheltered by my family, I just felt the need to venture out and connect with different folks from all walks of life. So, I wanted to jump into work early and gather tons of life experiences along the way [D3-7-7].*

This phenomenon—the strong desire for autonomous control over their lives, and the intention to set the course for a promising future (Barhate & Dirani, 2022)—reflects another source of young workers’ career motivation during the career establishment phase.

### 3.3. Career Growth Model: Psychological Mechanisms Promoting Career Striving

**Figure 1:**
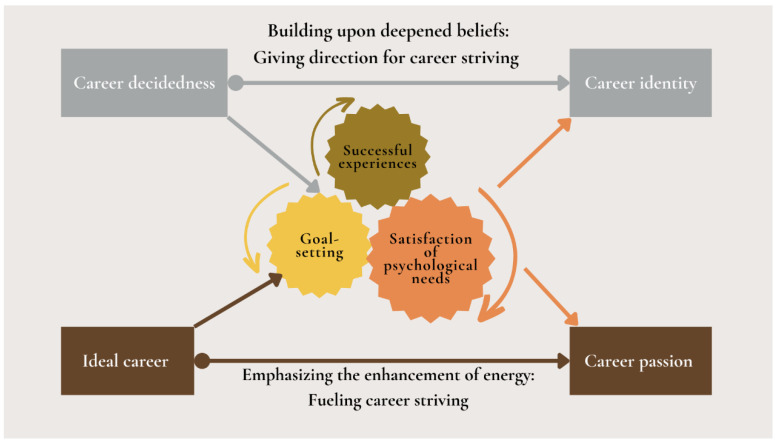
Career growth model.

A “Career Growth Model” was formulated from the self-determination perspective of basic psychological needs (Ryan & Deci, 2017) to explain career striving. The model explains the interplay between goal setting, successful experiences, psychological needs satisfaction, and the consequent promotion of career identity and passion during career establishment (see Figure 1 for a detailed representation). Participants reported that recognizing their capabilities through task accomplishments fostered mastery and competence. Continuous learning and skill development were crucial for overcoming constraints, setting goals, and enhancing autonomy (Alacovska et al., 2021; Ge et al., 2023; Hakvoort et al., 2022; Huang et al., 2020). The model highlights how these mechanisms promote proactive behavior and self-management, motivating young workers to pursue career advancement and fulfillment. The themes supporting this model are detailed in Table 2.

#### 3.3.1. Theme #7: Career Striving Is Contingent upon Continuously Recognizing One’s Own Capabilities Through the Successful Experiences of Accomplishing Work Tasks and Career Goals

Many participants felt that their current selves were distinct from who they were in university [D2, E4]. Over time, their abilities and responsibilities grew, making their work more interesting [B1]. This, in turn, opened up more possibilities for career growth and advancement.

Interviewer: When facing tough times, what gives you the strength to overcome difficulties?

Participant: It’s that sense of accomplishment I get when I achieve my goals. I really need that feedback to feel satisfied [E5-7-3].

Participants’ capabilities were affirmed by feedback received from others, but their own beliefs regarding their abilities were even more significant [B1, D1]. Besides securing a salary to support themselves, the various work life events that unfold, whether expected or not, as well as assigned tasks, were opportunities to showcase their competence [B3, D4]. These moments allowed them to gain the approval of others, increase their autonomy, and develop a sense of mastery (Ryan & Deci, 2017) when confronting responsibilities [C4, E5]. Observing others’ accomplishments and realizing that they can achieve the same fostered confidence in their own capabilities [E5].

These successful experiences provided lasting insights into their significance in the workplace, giving a clear understanding of their impact [E3]. This newfound clarity helped them identify the best ways to exhibit their abilities [F1].

#### 3.3.2. Theme #8: Continuous Learning and Personal Growth Are Fundamental Aspects of Career Striving

Participants revealed a constant drive to learn new things [B3]. Even after just six months of working, Participant B1 felt as though he had been working for a very long time and had gained a wealth of knowledge.

Various abilities were required to accomplish tasks that some young workers may not have been exposed to before entering the workforce. The theoretical concepts learned at school may have also differed significantly from their workplace realities [A3, C2]. Consequently, they learned how to apply their previous knowledge to specific job-related situations in their current work environments [A5]. Overall, the fundamental aspects of career striving are continuous learning and personal growth (Hakvoort et al., 2022; Huang et al., 2020; Rowold & Kauffeld, 2008).

*The main thing for me is to improve my abilities in various areas! I want to increase my chances of staying indispensable, so I keep learning and studying. It’s not just about feeling uncertain or threatened; it’s more about wanting more opportunities for myself. I don’t want to be stuck as a primary school teacher forever; I aim to grow and get promoted. Besides, some senior teachers’ attitudes make me want to prove them wrong and show that I have more to offer, regardless of my age or experience. [A4-12-5].*

The participants are no longer inexperienced newcomers with limited experience in writing about their past on their resumes [B3]. Both their hard and soft skills are growing rapidly [D4].

#### 3.3.3. Theme #9: Career Striving Solidly Delineates a Differentiated Development Trajectory, with Traces to Follow from Higher Education to Working

The workplace provided these young workers with challenging opportunities to showcase their abilities, attain success, and assert themselves. It also presented occasions for comparison with their peers [A5, F4]. Through these interactions, they could gauge their relative positions, understand their limitations, and gain a broader perspective on workplace dynamics [A2].

When young workers gain a broader understanding of the workplace, they become more focused and purposeful early in their careers. Guided by their goals, participants worked toward integrating their existing conditions, resources, and experiences [C3, F5] while investing in personal development to access more opportunities [F3]. This strategic approach enabled them to progressively carve out a path for career growth.

*I’ll be motivated to do what I want, and I’m ready to put in more time than others to achieve my goals [E5-2-9].*

During career establishment, they exhibited diverse developmental patterns, signifying that each person approaches their career in a distinctive manner as influenced by their career striving. Even in similar occupations or working within the same organization, newly hired employees display variations in their psychological states. These disparities are not attributed solely to external circumstances but also to the impact of the vocational socialization process [E2, B2] experienced during the higher-education-to-work transition.

#### 3.3.4. Theme #10: Young Workers Persistently Explore the Actual Work Environment and Seek Better Possibilities for Themselves

Many workers entering the workforce consider their current job a step toward their ideal career path. With an increasing network of contacts and practical work experience gained in the workplace, participants began adopting a more open and pragmatic outlook on future opportunities [C1, E4].

These young workers gradually evolved from a state of aimlessness to a more focused and practical approach to exploring and developing their careers. This shift enabled them to find better-suited and achievable career paths. During this process, their subjective understanding of career success became more tangible [D2, C5]. Apart from objective measures of success such as salary and position, their daily work accomplishments and meaningful external feedback played a crucial role in shaping their personal definitions and goal setting for success [E1]. Unlike their vague and challenging-to-evaluate experiences at university, they now face the reality of determining how to improve themselves and find personal fulfillment in their daily work lives. This transformation gives rise to a sense of direction and satisfaction in their career development.

*If I don’t pass the exam again next year, I might consider changing my environment… Some schools first start with temporary positions and then open official ones, so there’s a chance. I might prefer to go to schools like that [A3-6-4].*

#### 3.3.5. Theme #11: Successful Experiences Are Affected by Setting Personal Goals and Engaging in Consistent Practice and Completion, Which Deepen Self-Confidence

Besides psychological fulfillment, successful experiences often stemmed from achieving specific goals that provide immediate feedback (C1, E4). These goals may have involved something young workers had not attempted before or something they initially did not possess (F2).

The resulting sense of achievement fulfills the need for competence, autonomy, and relatedness (D2, F4). The continuous cycle of goal setting and subsequent success further reinforced young workers’ desire to find fulfillment through their work, driving ongoing career growth.

*Through these goals, I feel that when they trust me, yes, if I gain their trust, they would be willing to communicate with me, and they would be open to making changes together. So, if my influence is recognized, I would feel affirmed… Therefore, in my sense of achievement or successful experiences, I notice something similar, and it boosts my confidence (D5-9-2).*

Interestingly, the young workers often initially focused on being responsible and diligently completing assigned work tasks without clear self-directedness or personal goals in the workplace (B3). However, with time and adaptation, a settling-in phase gradually occurred, and their personal preferences or values began to influence them (Klehe et al., 2021). Even within the same work environment, different young workers had distinct goals (A5, E1). This realization imbues work with more personal significance as perceived work characteristics (Nielsen et al., 2008) gradually take shape.

#### 3.3.6. Theme #12: Pursuing Self-Fulfillment and an Increasing Sense of Competence Drive Goal Setting During Transitions

In general, a sense of competence directly influences goals and subsequent success. The demonstration of career striving through satisfaction and reinforcement of competence continued to drive participants’ intentions to achieve personal growth and improvement during the early phases of their vocational careers (C1, E4). This positive and growth-oriented tendency can be explained by self-efficacy related to task completion and a broader sense of competence (Deci et al., 2017). As suggested by the self-determination theory (Ryan & Deci, 2017), a sense of competence not only instilled confidence in completing daily work tasks but also allowed them to dream and have deeper aspirations (B1, C1). It also fostered confidence in their abilities to realize their dreams or ideal careers, leading to meaningful directions and a series of goals (F4).

Furthermore, after autonomously setting and achieving goals during the transitional period, young workers projected their self-fulfillment and moved toward subsequent self-actualization goals.

Participant: Back then, I was a bit overconfident, feeling like there was nothing I couldn’t do. What things can’t I accomplish?

Interviewer: Is high self-confidence the result of liking what I do, or did successful experiences in role-playing have a significant impact?

Participant: Those successful experiences had a significant impact; I felt that I could replicate them (B1-11-2).

#### 3.3.7. Theme #13: The Desire for Autonomy Arises During the Transition and Drives the Pursuit of Personally Meaningful Goals After Entering the Workplace

Many participants mentioned that their prolonged educational journeys suppressed their desire to breathe freely (D4, B2). When ending their education journeys and beginning career development (i.e., the establishment of a vocational career), they attempted to break free from past restrictions and take control of their work and lives (D2). It is as if they wanted to demonstrate their autonomy as a sign of growing up (C1); they did not want to be dictated to, even if it meant going through hardships (B5, D3).

*It all comes down to doing things I love, having autonomy, feeling happy, and a sense of meaning. The sense of autonomy is more significant than competence, and competence is more important than other factors (B3-10-3).*

In this phase, goals have clear personal significance and contribute to an increased sense of autonomy. In particular, the autonomous nature of goals reflects a significant shift in young workers’ career establishment. Unlike in the past, when they studied to please others or lived without a clear direction, their actions now seem to resonate with their pursuit of autonomy (A5, E1). This marks a profound turning point in their attitudes toward life, which subsequently manifests in the pursuit of personally meaningful goals and further strengthens their satisfaction with and sense of success in seeking autonomy.

### 3.4. Stress Coping Model: Psychological Mechanisms When Facing Pressure

**Figure 2:**
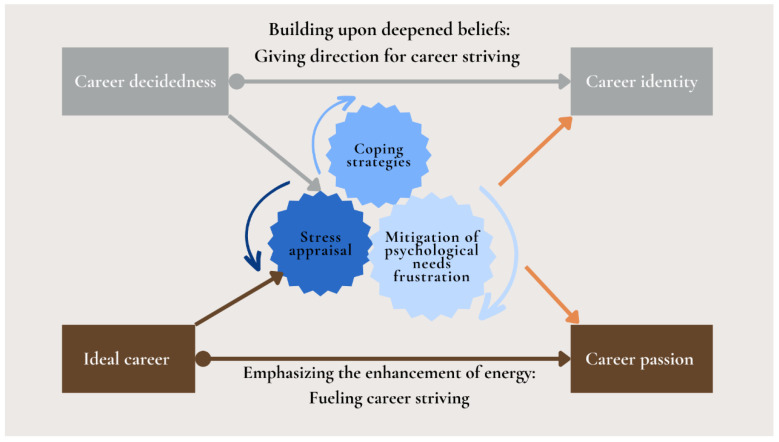
Stress coping model.

The stress-coping model based on the SDT framework (Holding et al., 2020; Ryan & Deci, 2017; Van Tuin et al., 2020) explains how young workers manage career stress. When examining the trajectories of career striving, it becomes imperative to elucidate the coping processes and experiences young workers undergo when facing pressure and how these experiences influence their resilience and continued development in the pursuit of their careers (Jiang et al., 2021). This model explains the mechanisms employed by young workers when confronting pressure, encompassing stress appraisal, coping strategies, and the mitigation of psychological needs frustration. (see Figure 2). Participants reported that coping with pressure and adapting to new work environments reshaped their self-concept. Balancing ideals with realities helped them overcome exhaustion and seek positive experiences. Support from social networks provided emotional relief and motivation. Reframing challenges as steps toward ideal careers reinforced their goals, while past successes fostered competence and proactive behavior, emphasizing adaptability and growth (Hirschi & Pang, 2025; Huang et al., 2020; S. Wang et al., 2021). These mechanisms contribute to continuous career striving during the establishment of a vocational path. The themes supporting this model are summarized in Table 3.

#### 3.4.1. Theme #14: Experiencing New Career Insights and Reshaping Beliefs Through Dealing with Pressure and Adaptation

A new understanding of oneself does not come from succeeding in the workplace, setting, or achieving goals. It also arises from how young workers handle pressure and adapt to their work environment. Similarly to having successful experiences, facing setbacks or failures at work may differ from encountering them at university and may provide a more realistic view of one’s capabilities and limitations in real work situations (Alacovska et al., 2021; Klehe et al., 2021; S. Yang et al., 2021).

Work challenges and career obstacles can lead to negative emotions such as frustration, anger, sadness, resentment, or loneliness (Kidd, 2004; Zapf et al., 2021). These pressures might originate from temporary work situations [C2] or an unfriendly work environment [F3, F5]. However, these new experiences helped young workers reevaluate themselves [E4], allowing them to determine whether they needed to adjust their previous beliefs. Experiencing failure and pressure helps young workers understand what they can and cannot change about themselves. This understanding led to them developing a more practical career self-concept that aligns with their current experiences and career goals.

*If I don’t achieve my goals, I might doubt my abilities and feel a sense of helplessness and disappointment… I feel that the work environment has a significant influence on who I am now. It provides an outlet for expressing myself, sharing my feelings, and even seeking support [C-2-9].*

#### 3.4.2. Theme #15: Developing Flexibility to Balance Ideals and Realities During the Process of Coping with Pressure

Many participants described how they adapted to the work environment and the challenges they experienced after entering the workplace, particularly regarding quickly feeling mentally exhausted [A2, F1]. Balancing reality and ideals became a tug-of-war while striving to adjust to exhaustion and mental strain [E3, D5]. Encountering poor treatment in the work environment was essential for them as it motivated them to seek positive experiences and regain the strength to move forward [A5, F2].

When facing circumstances that brought about negative emotions, they drew from past experiences to understand their current adversities. Reflecting on these experiences helped them develop the ability to navigate between their ideals and realities.

*Live in the present, enjoy the moment! When something happens, I try to understand and solve it. I used to question my abilities and whether I could do it, but now I just go ahead, give it a try, and then adjust if needed [D4-2-9].*

Young workers who tended to reflect on their past successful experiences and seek positive experiences seemed more willing to believe in themselves, even during challenging times. They confronted their current unsatisfactory states by viewing their present experiences as changeable individual experiences rather than making absolute judgments [A2, E4].

#### 3.4.3. Theme #16: Social Networks Provide Support and Consultation for Coping with Pressure and Problem-Solving

When faced with stress in the workplace, existing social networks and organizational support systems became crucial in providing effective coping mechanisms.

*I tend to confide in friends and family when I encounter such difficulties or challenges. It helps me release the pressure and not keep everything bottled up. I used to keep things to myself, but now I can’t bear the feeling of suppressing it. I find comfort in talking to my bandmates. Being listened to is so important [B4-13-2]!*

Some work issues could be easily resolved through advice from experienced individuals, which helped improve their work abilities [B3]. However, many stressors are associated with unchangeable environmental conditions and interpersonal factors [C1, F4]. Seeking support or having an outlet for expression instantly eases negative emotions, as it provides a sense of commonality and reduces loss of autonomy, connectedness, and competence, thus preventing basic psychological needs frustration (Holding et al., 2020; Van Tuin et al., 2020; Vansteenkiste & Ryan, 2013).

Participants frequently mentioned the need to adapt, learn from others’ perspectives, and reflect on their coping strategies in response to pressure, allowing them to maintain their self-belief and motivation [B4]. Insufficient social support for coping with pressure can lead to negative thinking, confusion, and feelings of loss [C2]. Being stuck in such a psychological state hinders progress, making it challenging to identify potential opportunities for growth [F5].

#### 3.4.4. Theme #17: Understanding the Current Situation and Unchangeable Conditions Through Reframing or Gaining Additional Meaningful Perspectives

Young workers interpret difficult and unchangeable circumstances differently. Some participants emphasized their current experiences as stepping stones toward their ideal paths [A1], while others focused on the growth they gained from current work challenges, which helped expand the conditions and necessary abilities for their career development [C4, E1]. The common thread among them is their future-oriented perspectives on present difficulties, which was crucial in maintaining their goals and sense of hope during career establishment. This coping strategy provided another mechanism for career striving.

*I imagine myself now as if I am in the fundraising or children’s theater, running a company (this is the participant’s ideal career). There are many things that others haven’t done before, but I am willing to try, and I want to see what the outcome will be. These are all creative processes that others can’t do or haven’t thought of, but for me, it’s about creating something. Oh, this may be related to my future entrepreneurial endeavors. I feel like I am accumulating the necessary experiences for that [A1-14-2].*

#### 3.4.5. Theme #18: The Role of a Sense of Competence in Being Motivated to Face Difficulties and Setbacks in Stressful Situations

Regarding the frequency of the three basic psychological needs mentioned in the interview process, sense of competence appeared to be the most important and commonly addressed. This study revealed that one’s sense of competence is a key factor in determining whether they will behave proactively when facing setbacks or difficulties. Wanting to prove oneself capable amid setbacks becomes a crucial motivation driving career striving.

Those with a higher overall sense of self-efficacy in assessing their abilities and who frequently experience fulfillment from past growth experiences at various work environments tended to remain less anxious and applied their self-beliefs amid difficulties and setbacks. Throughout the process of overcoming challenges and coping with stress, they developed new areas of competence, which further solidified their self-beliefs, in turn influencing their career advancement efforts and showcasing more confidence and strength.

*Throughout my growth process, I firmly believed that I had the ability to overcome challenges. I have confidence in my own capabilities. [A1-7-4]*

#### 3.4.6. Theme #19: Persisting Beliefs Derived from Positive Feedback and Confidence Gained Through Overcoming Adversity in Past Growth Experiences

Regardless of learning, personal life, or career situations, the positive feedback received from overcoming challenges resonates within individuals and is internalized as self-confidence when facing external circumstances. The participants’ beliefs in their abilities to surmount their current career- or job-related difficulties (B5, E5) empowered them to withstand challenges. Even in the face of an unsupportive environment or “negative forces” (F3), they were certain of their capacity to counteract adversity and prevail.

*My future is within my grasp, and I refuse to give in… I could have left that environment; many in my class transferred schools, but I felt I didn’t want to, I didn’t want to… It’s like, it’s like feeling a little weak, but I believe I can overcome it all, conquer everything, even the negative forces. Back then, when I was so young, it was this refusal to accept defeat, this belief that I could endure—those thoughts were there long before. (B1-5-4)*

Moreover, the ability to continuously surmount setbacks subtly influenced their competence self-evaluations. Turning the tide of discouragement, they learned that they can handle new or more difficult work assignments, which helped them become more ambitious and driven.

#### 3.4.7. Theme #20: Adaptability in Coping Reveals a Psychological Inclination for Career Crafting Through Effective Stress Management

Unlike passively complaining about unfriendly work environments, many participants realized and actively practiced the notion that individuals can change or shape their environments. This proactive and optimistic mindset allowed them to assert their vision for the future instead of reacting passively.

*I don’t give up; I try to improve myself and have the belief in creating something new [E5-10-6].*

*Yeah, I believe it’s not the environment, the world, or society that changes us; we have the opportunity to change it all… I think it’s about daring to pursue what we want… it’s gradually brewing [D5-9-6].*

Career crafting can be understood as actively creating opportunities to showcase one’s abilities and characteristics (Akkermans & Tims, 2017; Ge et al., 2023; Jiang et al., 2021; S. Wang et al., 2021). This adaptive and proactive psychological state reflects a shift in the individual–environment fit perspective. That is, rather than passively coping or adapting to the work environment and its challenges, some young workers adopt a proactive approach. They create a work environment that suits their career development paths and gain fulfillment and satisfaction, exhibiting a more active and dynamic orientation toward achieving person–environment fit.

## 4. Discussion

Career identity provides direction for career development, whereas career passion supplies the energy required for it. These, in turn, influence the extent to which young workers manage their careers amid the uncertainties that accompany contemporary careers. Furthermore, in career striving, young workers view these challenges as opportunities for self-growth that empower them to proactively pursue long-term career development and shape their trajectories toward career fulfillment.

### 4.1. Toward the Establishment of Career Striving Theory

The findings of this study provide a robust foundation for theorizing career striving as a multidimensional and developmental process grounded in both identity formation and motivational dynamics. Rather than viewing career striving as a fixed trait or a simple behavioral outcome, the study reveals it to be a psychologically complex and evolving construct that is dynamically shaped across the stages of early career establishment. The data gathered through in-depth interviews and structured coding across three stages of career development (pre-graduation, transition, and early work experience) provides insight into how young adults generate, refine, and sustain their career pursuits.

Career striving begins with a foundation in career identity, which emerges from a combination of exploratory activities and early goal formation during higher education. However, this identity remains fluid, subject to change as young adults move through the threshold of entering the workforce. The findings indicate that participants often enter the workforce with unresolved or exploratory career orientations, which later evolve through practical application, reflection, and real-world constraints. This evolution reinforces the importance of adaptive identity development as a key process in striving (Bennett, 2021; Branje et al., 2021; Van der Hallen et al., 2024), where idealized visions of one’s future self become tempered by experience and recalibrated to align with personal meaning and realistic goals.

Parallel to the evolution of identity is the development and refinement of career passion. Initially fueled by aspirational visions and educational experiences, career passion is likely to become sustainable when it is supported by a workplace context that affirms young workers’ psychological needs (Coxen et al., 2021; Hashiguchi et al., 2021; Lataster et al., 2022). As recent researchers argued (Olafsen et al., 2025; Q. Zhang et al., 2022), a sense of competence derived from meaningful accomplishments, opportunities for autonomy, and a supportive relational environment were shown to be essential for preserving and intensifying passion. Without these supports, passion diminishes, and workers may struggle to maintain engagement or persistence in the face of obstacles.

Moreover, the study reveals that psychological mechanisms driving career striving are not limited to positive reinforcements. A major contribution of this study is the demonstration that pressure, setbacks, and coping responses can equally shape developmental outcomes. When young adults are confronted with workplace stressors, their responses, ranging from reframing challenges to seeking social support, act as catalysts for self-transformation (Auletto, 2021; Hakvoort et al., 2022; S. Yang et al., 2021). Those who develop meaning-focused coping strategies report increased clarity of career direction, renewed motivation, and a deeper connection to their vocational identity (Ji et al., 2022; Patillon et al., 2025). As van Hooff and van Hooft (2023) have highlighted, these findings suggest that coping mechanisms are not simply reactive tools but are integral to the active construction of career striving trajectories.

These mechanisms interact in complex ways. Identity provides direction and coherence, passion fuels persistence and enthusiasm, and coping strategies convert challenges into developmental momentum. Career striving, therefore, is best understood not as a linear process, but as a recursive and context-sensitive interplay among motivational, cognitive, and relational factors. The evidence across the three tables (i.e., evolution of striving through stages, mechanisms promoting growth, and mechanisms under pressure) illustrates a coherent, layered understanding of how striving is both generated and sustained (Curşeu et al., 2021; Donald et al., 2020; Ge et al., 2023).

The role of social relatedness further nuances this picture. While autonomy and competence were consistently identified as critical psychological needs, the influence of relatedness varied according to the occupational context. In professions requiring extensive collaboration, supportive relationships were central to reinforcing self-efficacy and maintaining motivation (Auletto, 2021; Chami-Malaeb, 2022; Lipscomb et al., 2022; Musenze et al., 2021; Retkowsky et al., 2023; Stremersch et al., 2021). In other cases, relationships provided the emotional scaffolding that helped participants make sense of their experiences and stay committed to their goals. This difference highlights that the impact of social context is not uniform but must be interpreted within the interplay of individual disposition and work environment.

The theoretical contribution of this study lies in its integrated framework that reconceptualizes career striving as a dynamic process shaped by identity, passion, and meaning-making under both opportunity and constraint. The findings challenge reductive views of striving as a function of goal-setting or persistence alone (De Clercq et al., 2022; Huang et al., 2020; Greco & Kraimer, 2020), suggesting instead that striving is a reflective, emotionally engaged, and socially situated phenomenon. This model deepens our understanding of how young workers actively construct their vocational life and provides a template for future research to explore its applicability across diverse populations and cultural contexts.

Practically, the insights derived here hold significance for organizations, educators, and policymakers. Training programs that address both career skills and emotional regulation, coaching interventions that emphasize meaning-making, and policies that promote decent work conditions (Blustein et al., 2023; Graça et al., 2021; Otu, 2024; Zapf et al., 2021) are all strategies that can cultivate career striving. For educators, the implication is to go beyond employability training and include reflective practices that strengthen identity development and passion articulation (Avargil et al., 2025; Ho et al., 2021; Rath, 2025; X. Zhang et al., 2025).

Altogether, the study affirms that career striving is not merely about ambition or planning (Hirschi & Spurk, 2021). It is a complex, evolving process embedded in personal meaning, shaped by relational contexts, and catalyzed by both successes and struggles. This expanded view contributes to a more holistic understanding of career development in contemporary contexts and provides a useful lens for enhancing both theory and practice in human resource development and career counseling.

### 4.2. Limitations and Strategies to Address Them

While this is the first attempt to examine career striving using career identity and passion trajectories throughout career establishment, and much effort has been devoted to enhancing the trustworthiness of the data, the results should be interpreted with caution owing to several key limitations. These limitations include a small sample size, potential risk of researcher bias in interpreting and analyzing the data, and limited applicability or generalizability to different cultural contexts.

The small sample size restricts the generalizability of findings, and thus, future studies should aim for larger, more diverse participant pools to enhance the applicability of results across various cultural and occupational contexts. To mitigate the potential researcher bias in qualitative analysis, employing a team-based approach to data coding and interpretation, along with member checks, can ensure a more objective and validated representation of participants’ career striving experiences. Future studies should incorporate cross-cultural comparisons to explore how different cultural norms influence career identity and passion, thereby enriching the theoretical framework of career striving. By addressing these limitations, future research can build on the foundational insights of this study while providing more robust, applicable findings that better serve young workers navigating their career trajectories.

### 4.3. Future Directions for Exploring the Mechanisms Behind Career Striving

Future research should explore young workers’ trajectories in managing and striving for career development throughout their education-to-work transitions, as well as the dynamics underlying this evolution in the early years of entering the workplace (Auletto, 2021; Schoon & Heckhausen, 2019). The future research directions are as follows:

#### 4.3.1. Differentiating the Determinants, Functions, and Consequences of Self-Dialogue and Dialogue with Others in Career Striving

This study showed that the process of career construction often occurs during periods of change or adversity (Kutscher & Mayrhofer, 2023; S. Yang et al., 2021) and involves how young workers perceive themselves, find new equilibria, and develop new self-awareness by interpreting events and experiences. It demonstrated that young workers exhibit distinct tendencies in self-construction and social construction within various work contexts. However, as social constructionism argues, the main mechanisms underlying career construction involve self-dialogue and dialogue with others (Del Corso & Rehfuss, 2011; Patton, 2019; P. Yang & Chen, 2024; Young & Collin, 2004). These mechanisms are essential sources of motivation for career growth and striving in career establishment. Therefore, it is important to differentiate between the determinants, functions, and consequences of young workers’ self-dialogues and dialogues with others in career striving.

#### 4.3.2. Focusing on Transforming Needs Frustration into Needs Satisfaction

It is important to note that the mechanisms behind career striving, which are unclear in this study, involve transforming need frustration into psychological need satisfaction (Van Tuin et al., 2020; Vansteenkiste et al., 2020). Although Vansteenkiste and Ryan (2013) preliminarily discussed this issue, the rationale underlying this transformation remains unknown. Advancing knowledge in this area is essential to deeply understanding career striving. Related to this issue, it would be interesting to clarify how young workers draw strength from past failures, leading to enduring personal beliefs that provide stability and confidence during challenges.

## 5. Conclusions

This study examined career striving by linking career identity and passion to demonstrate the proactive nature of career self-management. It explored the formation of career identity and passion and tracked the trajectory of career striving from pre-graduation career preparation to entering the workforce and the early years of working. This study provides insights into how the interplay between the individual and the environment influences the development of vocational pathways among contemporary young workers, as contextualist career explanations (e.g., Chen, 1998; Fantinelli et al., 2023; Young et al., 2002) emphasize. Evidently, many complex mechanisms and psychological processes involved in career establishment still require further clarification. Future research should prioritize understanding the determinants, functions, and consequences of career striving, supported by rigorous qualitative and quantitative designs to establish a more comprehensive career striving theory.

## Figures and Tables

**Table 1:** How career striving evolves across developmental stages.

Career Development Stage	Thematic Dimension	Theme	Key Findings
Stage 1: Pre-graduation career preparation	Direction of career striving: Evolving career identity	Theme 1: Career direction is still being explored during university and continues into early work life	Career exploration activities in university support self-understanding, but are insufficient to form a clear, stable career direction at graduation.
	Motivation for career striving: Emerging career passion	Theme 4: Career passion originates from an idealized career vision during higher education	Passion formed in the university context motivates future vocational pursuits and serves as an early driver of career striving.
Stage 2: Transition into the workforce	Direction of career striving: Evolving career identity	Theme 2: Pre-professional identity evolves as university-acquired knowledge and skills are applied in the workplace	The integration of learned knowledge and early work experience contributes to the development of a vocational self-concept and evolving career identity.
	Motivation for career striving: Strengthening career passion	Theme 5: Practical success reinforces the pursuit of ideal career goals	Early work achievements boost self-confidence and strengthen motivation to pursue career establishment.
Stage 3: Early career establishment	Direction of career striving: Stabilizing career identity	Theme 3: Career identity crystallizes through compromise between ideals and workplace reality	Young workers develop more functional and realistic career goals by reconciling early aspirations with real-world constraints.
	Motivation for career striving: Persistent career passion	Theme 6: Striving is fueled by a desire to break free from past limitations	Motivation is reinforced by a need to overcome familial or educational constraints, propelling continued career development and striving.

**Table 2:** Career growth model: Psychological mechanisms promoting career striving.

Psychological Mechanisms Promoting Career Striving	Theme	Key Findings
Self-recognition through accomplishments	Theme 7: Career striving is contingent upon continuously recognizing one’s own capabilities through the successful experiences of accomplishing work tasks and career goals	Successful work experiences affirm young workers’ capabilities and foster a sense of mastery.
Drive for learning and development	Theme 8: Continuous learning and personal growth are fundamental aspects of career striving	Young workers exhibit a strong drive for continuous learning and skill development, as essential for career advancement.
Developmental trajectory across stages	Theme 9: Career striving solidly delineates a differentiated development trajectory, with traces to follow from higher education to working	Career striving is shaped by workplace experiences that provide opportunities for self-assertion and peer comparison.
Exploratory goal orientation	Theme 10: Young workers persistently explore the actual work environment and seek better possibilities for themselves	A proactive exploration of career opportunities helps young workers define their career paths and measures of success.
Goal setting and consistency	Theme 11: Successful experiences are affected by setting personal goals and engaging in consistent practice and completion, which deepens self-confidence	Setting and achieving personal goals fosters a cycle of success that builds confidence and reinforces motivation.
Pursuit of fulfillment and competence	Theme 12: Pursuing self-fulfillment and an increasing sense of competence drive goal setting during transitions	A sense of competence influences the setting and achievement of personal and professional goals.
Autonomy development	Theme 13: The desire for autonomy arises during the transition and drives the pursuit of personally meaningful goals after entering the workplace	The transition to the workforce ignites a desire for autonomy, shaping the pursuit of personal goals.

**Table 3:** Stress coping model: Psychological mechanisms under pressure.

Psychological Mechanisms When Facing Pressure	Theme	Key Findings
Insight development under pressure	Theme 14: Experiencing new career insights and reshaping beliefs through dealing with pressure and adaptation	Coping with pressure leads to new self-insights and realistic self-concepts.
Balancing ideals and reality	Theme 15: Developing flexibility to balance ideals and realities during the process of coping with pressure	Young workers learn to manage between their ideals and workplace realities, enhancing their coping strategies.
Social support and consultation	Theme 16: Social networks provide support and consultation for coping with pressure and problem-solving	Social support systems are vital for coping with workplace stress.
Cognitive reframing	Theme 17: Understanding the current situation and unchangeable conditions through reframing or gaining additional meaningful perspectives	Reframing challenges as growth opportunities supports career striving.
Sense of competence in adversity	Theme 18: The role of a sense of competence in being motivated to face difficulties and setbacks in stressful situations	A strong sense of competence drives proactive behavior in the face of challenges.
Confidence from past growth	Theme 19: Persisting beliefs derived from positive feedback and confidence gained through overcoming adversity in past growth experiences	Positive feedback reinforces self-confidence and resilience against adversity.
Coping and career crafting	Theme 20: Adaptability in coping reveals a psychological inclination for career crafting through effective stress management	Proactive coping strategies facilitate career crafting and personal fulfillment.

## Data Availability

The data that support the findings of this study are available from the author, but restrictions apply to the availability of these data, and they are not publicly available. The data are, however, available from the author upon reasonable request and with the permission of the Human Research Ethics Committee.

## Appendix A. Interview Guide and Sample Interview Questions

1. Pre-graduation career preparation:
  Can you describe your career goals during your final year of university?
  How did you prepare for your career while still in higher education?
  What kinds of proactive steps did you take during university to prepare for your future career (e.g., internships, networking, extra training)?
  How did you stay motivated or sustain your efforts while managing academic and career-related demands?
2. Transition to workforce:
  What were your experiences transitioning from university to your first full-time job?
  How did your expectations match the reality of your first job?
  In what ways did you take initiative or act proactively during the transition to help yourself adjust or succeed in your new role?
  Were there any steps you took to shape your opportunities or overcome initial challenges when starting work?
3. Early career experiences:
  Can you describe a challenging situation you faced in your first job and how you dealt with it?
  How have your career goals evolved since you started working?
  What proactive behaviors have you demonstrated to advance or reshape your career in your current role or workplace?
  Can you provide an example of a proactive step you took to advance your career?
  How do you maintain your career motivation or continue striving for long-term goals during this stage?
4. Career identity and passion (reflective cross-stage themes)
  How do you perceive your career identity now compared to when you first graduated?

What role does passion play in your current job and overall career development?

In the semi-structured interview process, the emphasis was on the participants’ subjective experiences, allowing them to freely narrate their career striving experiences through the interviewer’s use of these questions. The interviews were not conducted in a rigid, question-by-question manner.
